# Estrogen receptors alpha and beta in rat placenta: detection by RT-PCR, real time PCR and Western blotting

**DOI:** 10.1186/1477-7827-4-13

**Published:** 2006-03-28

**Authors:** Maie D Al-Bader

**Affiliations:** 1 Department of Physiology, Faculty of Medicine, Kuwait University, P.O. Box 24923, Safat, Zip Code 13110, Kuwait

## Abstract

**Background:**

High levels of estrogens during pregnancy not only retard placental and fetal growth but can lead to reproductive tract abnormalities in male progeny. Estrogens act through estrogen receptors (ER) to modulate the transcription of target genes. These ER exist in two isoforms, ER alpha and ER beta and recently several variants of these isoforms have been identified.

**Methods:**

The expressions of ER isoforms and variants have been studied in rat placenta at 16, 19 and 21 days gestation (dg). Gene expression was assessed using RT-PCR and real time PCR while protein expression was studied using Western blotting followed by immunodetection. Placental homogenates were probed with: a monoclonal antibody raised against the steroid binding domain of the ER alpha (ER alpha -S), a monoclonal antibody raised against the hinge region of ER alpha (ER alpha -H) and a polyclonal antibody raised against the amino terminus of ER beta.

**Results:**

ER alpha and ER beta mRNA and protein were detected from as early as 16 dg. Two PCR products were detected for ER alpha, one for the wild type ER alpha, and a smaller variant. Real time PCR results suggested the presence of a single product for ER beta. The antibodies used for detection of ER alpha protein both identified a single 67 kDa isoform; however a second 54 kDa band, which may be an ER alpha variant, was identified when using the ER alpha -H antibody. The abundance of both ER alpha bands decreased significantly between 16 and 19 dg. As for ER beta, four bands (76, 59, 54 and 41 kDa) were detected. The abundance of the 59 and 54 kDa bands decreased significantly between 16 and 19 dg.

**Conclusion:**

This study shows that both ER protein isoforms and their variants are present in rat placenta. The decrease in their expression near parturition suggests that the placenta may be relatively unresponsive to estrogens at this stage.

## Background

Placental growth and function are of biological significance in that placental tissue promotes prenatal life and pregnancy maintenance. In many mammalian species the placenta produces estrogens during pregnancy suggesting a role for placental estrogens as paracrine factors in the regulation of placental growth and differentiation [1]. However, in contrast to the human placenta, which plays a role in producing progesterone and estrogen, the rat placenta does not produce estrogen [2,3] and secretes only small amounts of progesterone [4]. Indeed the rat placenta is the principal source of testosterone in the peripheral circulation between days 14–18 of pregnancy [5] and this testosterone serves as the substrate for estradiol synthesis in the corpus luteum [6]. Thus although the rat placenta does not produce estradiol it sustains the ovarian production of this hormone.

Estrogen and progesterone are both essential for the initiation and maintenance of pregnancy in the rat. With regard to progesterone, growth and development of the embryo and fetus are unaffected over a wide range of progesterone concentrations in maternal plasma [7]. On the other hand, pharmacological doses of estrogens cause high levels of embryonic mortality [8], although administration later in pregnancy causes retarded fetal growth with less effect on survival [9].

In order for estrogens to exert their biological effects, they need to bind to the ER which then undergoes a conformational change allowing it to interact with chromatin and to modulate transcription of target genes [10]. ER exists in two different isoforms, ER alpha and ER beta (ERα and ERβ) [11-13]. Both proteins are physiologically relevant, they bind estradiol with high affinity and activate transcription of estrogen responsive reporter gene constructs expressed in mammalian cell lines [11,14].

There is increasing concern over the known effects of various chemicals released into the environment on the reproduction of humans and other species. It is well known that gonadal steroids function as organizational agents during fetal development and thus exposure of the developing fetus to exogenous estrogens can cause long term deleterious effects. This led to the assumption that a possible cause for the rise in male reproductive tract abnormalities may be the inappropriate exposure to estrogens or suspected environmental estrogenic chemicals during fetal and/or neonatal life [15,16]. The estrogenic potency of industrial-derived estrogenic chemicals is limited but the estrogenic potency of phytoestrogens is significant, especially for ERβ and may trigger many of the biological responses that are induced by the physiological estrogens [17].

The present study was therefore designed to: 1) investigate whether ER proteins are present in rat placentae and 2) to test the hypothesis of whether maternal estrogens act as local regulatory factors on ER mRNA and protein expression. Accordingly, estrogen receptor alpha and beta mRNA and protein expression was studied in rat placentae at different days gestation. In this article we have demonstrated that both ER isoforms (ERα and ERβ) are expressed in rat placentae as early as 16 dg and that these isoforms and some of their variants (term used in this article to refer to the subtypes of each isoform) tend to decrease with progression of pregnancy probably due to the high levels of sex steroids towards term.

## Materials and methods

### Materials

The polyclonal antibody corresponding to amino acids 1–150 of the ERβ was purchased from Santa Cruz Biotechnology, Inc., U.S.A (catalogue # H-150; sc-8974). Two monoclonal antibodies raised against different epitopes of the ERα were used: one raised against the steroid binding domain of the ERα (amino acid residues 495–595; referred to as ERα-S; catalogue # C-311; Santa Cruz Biotechnology, Inc., U.S.A) and the other raised against the hinge region (amino acid residues 287–300; referred to as ERα-H; catalogue # SRA-1000; Stressgen Biotechnologies Corp., Canada). The actin monoclonal antibody was purchased from Santa Cruz Biotechnology, Inc., U.S.A (catalogue # C-2). PVDF membranes were obtained from Amersham Pharmacia Biotech Ltd., U.K.

### Animals

Sprague-Dawley rats were obtained from Bantin and Kingman (U.K.). The rats were housed in the Animal Resources Centre at the Faculty of Medicine, Kuwait University and had free access to food and water. The rats were maintained on a cycle of 12 h light and 12 h darkness at 22°C. The experiments were carried out in accordance with the rules of laboratory animal care in this institution.

### Tissue collection

Female rats were mated with males and mating was verified by the presence of sperm in the vaginal smear; this was designated as day 0 of pregnancy. Pregnant dams were stunned and killed by cervical dislocation at 16, 19 or 21 days gestation (dg). Uterine horns containing conceptuses were removed and placed immediately on ice. Fetuses and placentae were separated and placental tissues from each litter were pooled (four pregnancies were obtained at each gestational age [n = 4] and three to four placental tissues per pregnancy were pooled). The maternal uterus and maternal brain of the 21 dg dams were used as a positive control (data not shown). The cryoprotective agent, dimethylsulfoxide, (10% v/v) was added before freezing at -70°C for subsequent analysis.

### RNA isolation and quantification

Samples were homogenized in denaturing solution (4 M guanidine thiocyanate salt, 25 mM sodium citrate, pH 7.0, 0.5% w/v sarcosyl and 0.1 M 2-mercaptoethanol) using a sterile hand-held homogenizer. To 3.6 ml homogenate, 0.36 ml sodium acetate (2 M, pH 4.0), 3.6 ml citrate buffer-saturated phenol (pH 4.3) and 0.72 ml chloroform:isoamyl alcohol (49:1) were added sequentially, shaking well between each addition. Tubes were vigorously shaken for 15 s after the final addition. Tubes were kept on ice for 15 min, then centrifuged (10,000 g for 20 min at 4°C). The aqueous phase was removed, avoiding the DNA interphase, and an equal volume of ice-cold isopropanol was added. After shaking, tubes were kept at -20°C for > 1 h. The precipitate was collected by centrifugation (10,000 g for 20 min at 4°C) and dissolved in 0.3 ml denaturing solution. Nucleic acid was re-precipitated by adding an equal volume of ice cold-isopropanol. After > 1 h at -20°C, the samples were centrifuged (10,000 g for 10 min at 4°C). The pellet was washed twice with 1 ml 75% (v/v) ethanol (at -20°C) by suspension/centrifugation. The final pellet was air-dried, then dissolved in 0.5% (w/v) SDS (0.25 μl/mg wet weight tissue) at 65°C for 15 min. Extracted RNA was stored at -70°C. The quality and quantity of total RNA sample was determined using spectroscopic measurements at 260 and 280 nm. Samples with A_260_/A_280 _ratios > 1.7 were only studied further. The integrity of total RNA was checked by agarose gel electrophoresis and 28S and 18S rRNAs visualized after ethidium bromide staining.

### Reverse Transcription Polymerase Chain Reaction (RT-PCR)

SDS was removed from total RNA by precipitation with sodium acetate-isopropanol then resuspended (at *ca*. 1 μg/μl) in water. The RNA concentration was determined by spectrophotometery and was adjusted to 0.5 μg/μl with water. All samples were DNase treated before reverse transcription. Briefly, 2 μg of total RNA was mixed on ice with 40 U of RNasin, 1 U of DNase and 1× DNase buffer in a final volume of 20 μl. The mixture was left at room temperature for 15 min and the reaction was terminated by adding 2 μl of 25 mM EDTA and heating at 70°C for 10 min. The DNase-treated sample was divided into two 11 μl aliquots, 100 ng random hexamers were added and the mixture was heated at 70°C for 10 min, immediately chilled on ice for > 3 min then briefly centrifuged. With the tube on ice, the following were added: 1× first strand buffer (25 mM Tris-HCl pH 8.3, containing 37.5 mM KCl and 1.5 mM MgCl_2_), 5 mM DTT and 500 μM dNTP mix. To one tube (RT+ reaction) 200 U Superscript II RNase H^- ^reverse transcriptase were added, whereas water was added to the other tube (control RT-reaction) in a final volume of 20 μl. After gentle mixing, reactions were incubated at room temperature for 10 min then at 42°C for 50 min. Reactions were terminated by heating at 70°C for 15 min.

The PCR reaction was carried out in a programmable thermal cycler (Perkin Elmer, model 9700). The ERα primer sets used were rERα U (5'-TAAGAACCGGAGGAAGAGTTG) and rERα L (5'-TCATGCGGAATCGACTTG) that give an expected 623 bp product. The 18S housekeeping gene primer sets r18S U (5'-GTCCCCCAACTTCTTAGAG) and r18S L (5'-CACCTACGGAAACCTTGTTAC) give an expected 419 bp product. The reaction mixture consisted of: 1× PCR buffer (20 mM Tris/50 mM KCl), 3 mM MgCl_2_, 0.5 mM dNTPs and 0.3 μM each of upper and lower primers, 0.5 μl template (RT^+^, or RT- in addition to a water sample used as a negative control) and 1.25 U recombinant Taq DNA polymerase in a final volume of 25 μl. The PCR reactions were then cycled as follows: 5 min at 94°C (1 cycle); 30 s at 94°C (denaturation step), 30 s at the 45°C (annealing step) and 1 min at 72°C (extension step) for the required number of cycles (24 cycles for 18S and 32 cycles for ERα). Tubes were then incubated for a further 7 min at 72°C (1 cycle).

### Real Time PCR (ReT-PCR)

The ReT-PCR reaction was carried out in a ReT-PCR system (Applied Biosystems, model 7500). The endogenous control, β-glucuronidase, used in this experiment was supplied by Applied Biosystems. The ERβ primer sets used detect both the wild type and a variant that has a 54 bp (18aa) in-frame sequence between exons 5 and 6 of wild type ERβ [18]. These primer sets were rERβ LBD U (5'-GAGCTCAGCCTGTTGGACC) and rERβ LBD L (5'-GGCCTTCACACAGAGATACTCC) [19]. The PCR reactions were prepared using the Taq Man universal master mix (# 4324018, Applied Biosystems) then cycled as follows: 2 min at 50°C (1 cycle); 10 min at 95°C (1 cycle), 15 s at 95°C and 1 min at 60°C for 60 cycles.

### Isolation of proteins and western blot analysis

Placentae were thawed and washed twice with ice-cold isotonic saline then homogenized in ice-cold homogenization buffer containing glycerol [to stabilize the receptors] and protease inhibitors (10 mM Tris-HCl pH 7.4, 1.5 mM EDTA and 10% v/v glycerol, 1.0 mM DTT, 1 μg/ml leupeptin, 100 μg/ml bacitracin, 2 μg/ml aprotonin, 1 μg/ml pepstatin A), using a Polytron homogenizer, to yield a 5% (w/v) homogenate (the total receptor fraction). Homogenates were passed through a nylon mesh (110 μm pore size) then through steel gauze (40 μm pore size). An aliquot was removed for protein assay. Samples (50–100 μg protein) and molecular weight rainbow markers (14,300 – 220,000 daltons; Amersham Pharmacia Biotech Ltd., U.K.) were mixed with loading buffer (0.03% w/v bromophenol blue, 32 mM Tris-HCl pH 6.8, 8% w/v dithiothreitol and 4% w/v SDS), boiled for 5 min, then chilled on ice and centrifuged (10,000 × g for 1 min). Samples were then electrophoresed (SDS PAGE; 7.5% polyacrylamide gels). The distance migrated by the marker proteins and bromophenol blue front were measured. The buffer chamber of the electrophoresis apparatus was filled with degassed transfer buffer (25 mM Tris-HCl, 150 mM glycine, 20% v/v methanol and 0.1% w/v SDS, pH 8.3). Gels were equilibrated in transfer buffer then protein was transferred to PVDF membranes with a constant current of 300 mA overnight with cooling. After transfer the membranes were dried and stored at 4°C.

### Immunodetection

Membranes were blocked for 1 h at room temperature with 10% non-fat dry milk in TBS-T (20 mM Tris, 137 mM NaCl, pH 7.6 and 0.1% v/v Tween). The membranes were washed by rinsing twice with TBS-T, followed by two 10 min washes (20 ml/wash). Membranes were then incubated overnight at 4°C with 3 ml primary antibody solution diluted in 5% w/v non-fat dry milk in TBS-T (ERα-S diluted 1:500, ERα-H diluted 1:1000 and ERβ antibody diluted 1:1000). After incubation, the membranes were rinsed twice with TBS-T followed by one 15 min wash and two 5 min washes (20 ml/wash). Membranes were incubated with the appropriate secondary antibody (anti-mouse Ig HRP linked diluted 1:20000 or anti-rabbit Ig HRP linked diluted 1:10000 in TBS-T) for 1.5 h at room temperature then rinsed twice with TBS-T followed by one 15 min wash and four 5 min washes (20 ml/wash). Detection was by chemiluminescence after incubation with an HRP-linked secondary antibody – using a commercial kit (ECL-Plus; Amersham Pharmacia Biotech Ltd., U.K.) and standard methodology. After obtaining the results for the respective estrogen receptor primary antibodies, the membranes were stripped in stripping buffer (100 mM 2-mercaptoethanol, 2% SDS, 62.5 mM Tris-HCl pH 6.7) at 50°C for 30 min with occasional shaking. The membranes were then washed twice in TBS-T for 10 minutes each before being re-probed with anti-actin antibody (diluted 1:500 in TBS-T) which served as an internal control. All results were expressed relative to actin.

### Protein determination

Protein was determined by a dye-binding method using Coomassie Plus Protein assay reagent (Pierce & Wariner Ltd, U.K.)

### Determination of protein sizes

In order to determine the sizes of the bands a marker lane was always included. The distance migrated by the protein marker was measured and a standard curve constructed using a plot of log_10 _molecular mass versus relative mobility (distance migrated by band ÷ distance migrated by dye; R_f_). The size of the unknown protein band was then determined using the equation of the line.

### Data analysis

After the RT-PCR reaction for ERα, PCR products were electrophoresed alongside a 100 bp DNA marker (100 bp ladder, Gibco), through a 2% (w/v) low electroendosmosis (LE; Boehinger Manheim) agarose gel stained with ethidium bromide. All images were captured using Gene Genius Bio Imaging System and od values of PCR products were measured using Gene Tools Software. As for ReT-PCR the results for the average CT threshold cycle for each sample was used to express the relative expression of ERβ to β-glucuronidase.

Statistical analysis was performed using SPSS (ANOVA followed by post-hoc analysis [LSD]) when the test for homogeneity of variance was fulfilled and using Games-Howell post-hoc analysis when the homogeneity of variances was not attained. All data shown are mean ± S.E.M. and a p value of < 0.05 was taken as the minimum level of significance.

## Results

### Gross parameters

As expected there was a significant increase (p < 0.001) in placental weights and fetal body weights with gestation (Table 1). As for the litter number, there was a significant decrease (p < 0.001) between 16 and 21 dg and fetal resorptions were detected in some dams, however, these dams were not included in this study.

**Table 1:** Litter size, feto-placental growth and placental protein measurements.

**Days gestation (dg)**	**Litter number**	**Placentae weights (g)**	**Fetal body weights (g)**	**Protein concentration (mg/g wet wt)**	**Protein content (mg/tissue)**
**16**	12 ± 1	0.313 ± 0.010	0.513 ± 0.016	90.904 ± 3.559	28.489 ± 1.893
**19**	11 ± 1	0.508 ± 0.012*	2.371 ± 0.060*	98.004 ± 3.476	49.709 ± 1.014*
**21**	9 ± 1*	0.546 ± 0.007*	5.387 ± 0.166*	95.307 ± 3.713	52.053 ± 2.037*

Values are mean ± S.E.M. for four different dams. A significant increase (p < 0.001) in placental and fetal body weights during gestation and a significant decrease (p < 0.001) in litter number between 16 and 21 dg was detected. Placental protein concentration was measured using the Bradford method; a significant increase in protein content was observed between 16 and 19 dg and 16 and 21 dg (*p < 0.001; LSD test).

### Protein concentration and content

There was no significant change in placental protein concentration (Table 1), nevertheless, a significant increase in protein content was observed between 16 and 19 dg (p < 0.001). This increase was in parallel with the increase in placentae weight noticed with gestation (Table 1).

### Estrogen receptor alpha gene expression

**Figure 1:**
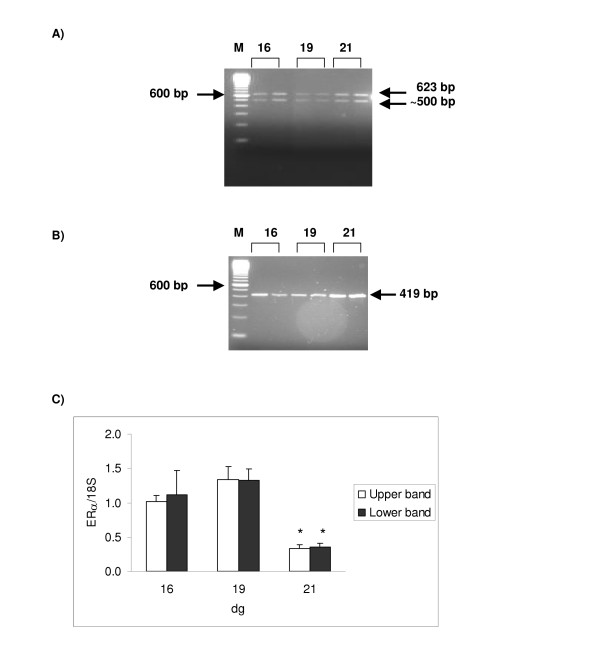
**Expression of ERα transcript in rat placentae during gestation**. [A] Ethidium bromide stained gel for ERα. The migration of the 100 bp marker (M) is shown on the left-hand side and the calculated size of the signal indicated on the right-hand side of the gel (expected size is 623 bp). An extra ~500 bp band was detected for ERα. Both RT- and water samples were negative (data not shown). Reactions were performed for 32 cycles at 45°C, using standard reaction conditions. [B] Ethidium bromide stained gel for 18S. The migration of the 100 bp marker (M) is shown on the left-hand side and the calculated size of the signal indicated on the right-hand side of the gel (expected size is 419 bp). Both RT- and water samples were negative (data not shown). Reactions were performed for 24 cycles at 45°C, using standard reaction conditions. [C] Relative ERα mRNA level. The amount of product was expressed relative to 18S. Samples were run in duplicates; results shown are mean ± S.E.M. (n = 4). A significant decrease in ERα mRNA expression was detected between 16 and 21 dg and 19 and 21 dg for both PCR products (*p < 0.001).

The expected PCR product (623 bp) for the ERα mRNA was detected in rat placentae from as early as 16 dg (Figure 1). However, a ~500 bp product was also detected from 16 dg. The RT- and W reactions did not show any bands (figures not shown), indicating that the amplified products in RT+ reactions are not due to genomic DNA nor due to contamination of reagents, respectively. The od measurements of PCR products obtained for ERα gene were expressed relative to the od obtained from 18S. When expressed relative to the 18S housekeeping gene it was seen that the pattern of expression of both products relative to 18S was similar with a significant decrease in ERα mRNA expression between 16 and 21 dg and 19 and 21 dg (*p < 0.001).

### Estrogen receptor beta gene expression

**Figure 2:**
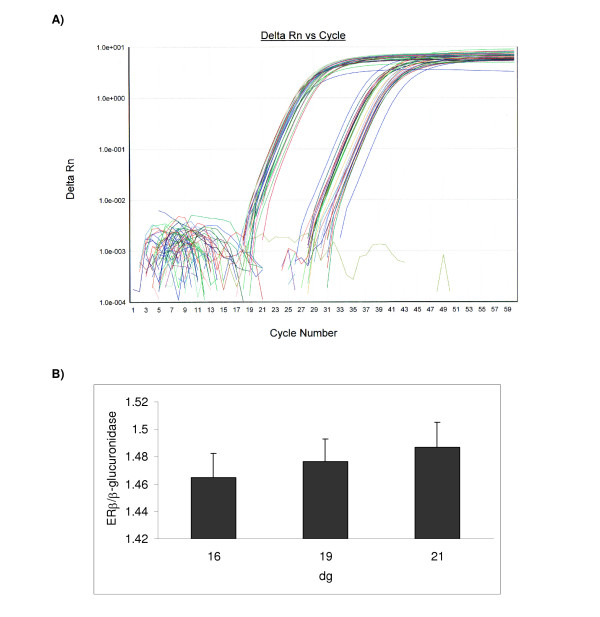
**Expression of ERβ transcript in rat placentae during gestation**. [A] ReT-PCR analysis for ERβ and β-glucuronidase. A graph showing delta Rn (change in normalized reporter expression) as a function of cycle number. Reactions were performed for 60 cycles at 60°C, using recommended conditions by supplier. Samples were run in duplicates for both target and endogenous genes. [B] Relative ERβ gene expression. The average threshold cycle (C_T_) for duplicate samples was expressed relative to that of β-glucuronidase. Results shown are mean ± S.E.M. (n = 4). No significant change in ERβ gene expression was detected.

Using RT-PCR it was not possible to detect ERβ mRNA expression. However, using ReT-PCR it was possible to amplify the ERβ gene (Figure 2). The average threshold cycle (C_T_) for duplicate samples was expressed relative to that of β-glucuronidase (endogenous or housekeeping gene). No significant change in ERβ gene expression was detected with gestation.

### Estrogen receptor alpha protein expression

**Figure 3:**
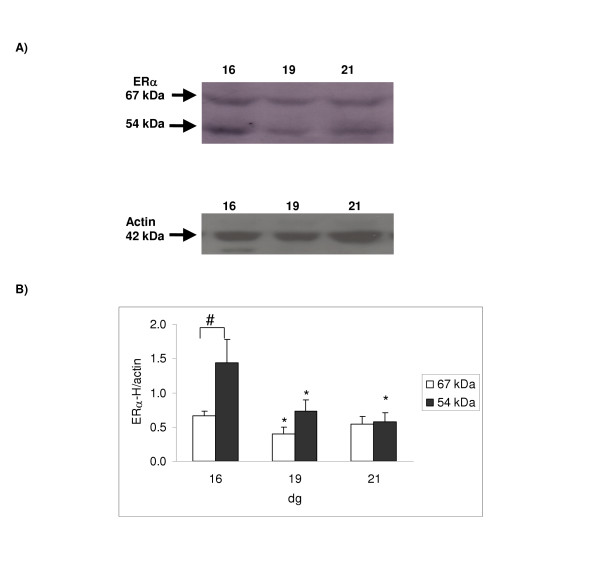
**Protein expression using a monoclonal antibody (ERα-H) raised against the ERα hinge region**. 100 μg of protein was loaded. [A] Representative Western blot for ERα and actin (which was used as an internal standard for normalizing the data); the calculated size of the band is indicated on the left-hand side of the gel. Two bands of apparent molecular weights of 67 and 54 kDa were detected for ERα. [B] Ontogenic profile: ERα protein was expressed relative to actin. Results shown are mean ± S.E.M. from four different dams; a significant decrease in ERα protein expression was detected between 16 and 19 dg for the 67 kDa band (*p < 0.05). For the 54 kDa band a significant decrease in ERα protein expression was detected between 16 and 19 dg and 16 and 21 dg (*p < 0.05). The 54 kDa band had a higher expression at 16 dg when compared to the 67 kDa band (#p < 0.001; LSD test).

**Figure 4:**
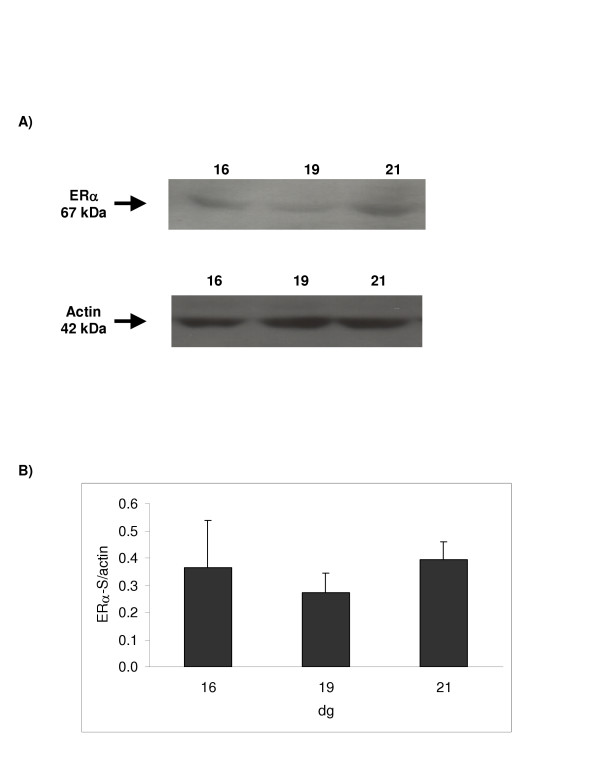
**Protein expression using a monoclonal antibody (ERα-S) raised against the ERα steroid binding domain**. 100 μg of protein was loaded. [A] Representative Western blot for ERα and actin (which was used as an internal standard for normalizing the data); the calculated size of the band is indicated on the left-hand side of the gel. [B] Ontogenic profile: ERα protein was expressed relative to actin. Results shown are mean ± S.E.M. from four different dams; no significant change in protein expression was detected.

A ~67 kDa single band for ERα was observed for the placental samples using both the ERα-H and the ERα-S antibodies from as early as 16 dg (Figures 3 &4, respectively). However, using the ERα-H antibody, another band of apparent molecular weight of 54 kDa was detected. The abundance of these ERα variants decreased significantly between 16 and 19 dg (p < 0.05) as shown when the ERα-H antibody was used. However, using the second antibody, ERα-S, no apparent change in protein expression was observed although the level at 19 dg appeared lower but did not reach significance. The 54 kDa band observed with the ERα-H antibody was more abundant at 16 dg compared to the 67 kDa (Figure 3). This difference in expression was normalized by 19 dg.

### Estrogen receptor beta protein expression

**Figure 5:**
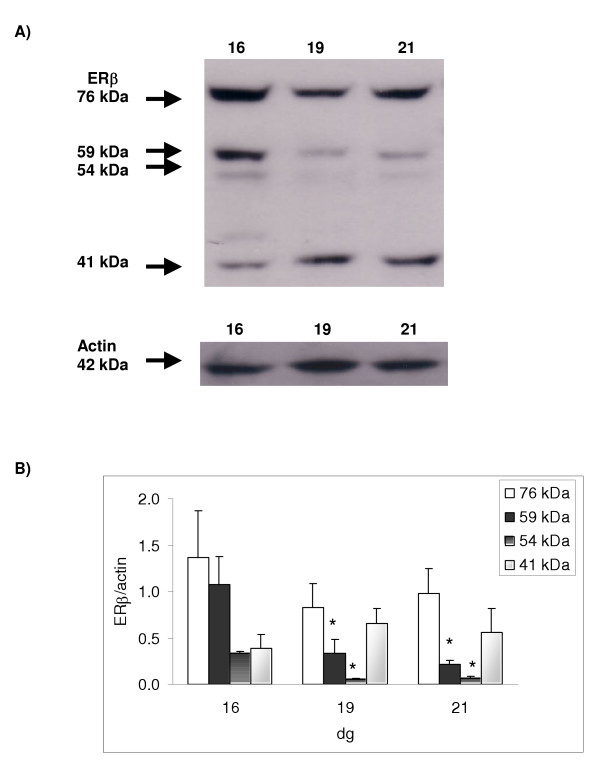
**Protein expression using a polyclonal antibody (ERβ) raised against the ERβ amino terminal**. 25 μg of protein was loaded. [A] Representative Western blot for ERβ and actin (which was used as an internal standard for normalizing the data); the calculated size of the band is indicated on the left-hand side of the gel. Four bands of apparent molecular weight of 76, 59, 54 and 41 kDa were detected for ERβ. [B] Ontogenic profile: ERβ protein was expressed relative to actin. Results shown are mean ± S.E.M. from four different dams; a significant decrease in ERβ protein expression (p < 0.001) was detected between 16 and 19 dg and 16 and 21 dg for both the 59 and the 54 kDa ERβ bands (LSD test).

When studying the expression of the second estrogen receptor isoform, ERβ, four bands were detected with apparent molecular weights of 76, 59, 54 and 41 kDa (Figure 5). When comparing the level of expression of the ERβ protein as pregnancy progressed, it was found that there was a significant decrease in expression of the 59 and 54 kDa bands (p < 0.001) between 16 and 19 dg. Moreover, the 76 kDa band showed a tendency to decrease at 19 dg, however, this was not statistically significant. On the other hand, the 41 kDa ERβ variant showed a different pattern of expression with a trend to increase between 16 and 19 dg (not statistically significant).

## Discussion

In this study dams were sacrificed at 16, 19 and 21 dg, the number of fetuses was determined, placentae were collected and ER status was verified both at the mRNA and protein levels. It was rather interesting to observe that as pregnancy advanced there was a reduction in litter size number. This decrease has been explained by other investigators, studying various animal models, as being possibly due to fetal resorptions, a reduction in the number of maturing follicles, and/or atrophy of ovaries [20,21]. In our study fetal resorptions were observed in some pregnancies, however, when this was the case these animals were excluded from the study.

The differentiation and growth of the rat chorioallantoic placenta occurs between days 8 through 21 and the expression of ER was studied between 16 and 21 dg. However, before studying the expression of the ER isoforms in placental samples, the protein concentration was determined. Placental protein concentration showed no significant change with gestation, however, the increase in placental weight was accompanied by an increase in protein content. This increase may have resulted from alterations in protein metabolism or cell gain.

It was clear when studying the expression of ERα and ERβ mRNA that there was a reduction in ERα mRNA by 21 dg; however, there was no change in ERβ mRNA expression with progression of pregnancy. A similar pattern for ERα and ERβ mRNA was reported in rat corpus luteum during pregnancy. The levels of ERα mRNA increased from early pregnancy, reached a maximum at mid-pregnancy and declined before parturition while levels of ERβ mRNA remained constant throughout pregnancy, with a significant decline at parturition [22]. Similarly a decline in ERα mRNA between days 11 and 16 of pregnancy was detected in rat decidua basalis [23].

To study whether the changes in gene expression reflect changes in protein, the expression of ERα and ERβ proteins were studied. For ERα, two antibodies were used, one raised against the hinge region of the ERα (ERα-H) and another against the steroid binding domain (ERα-S). It was found that when using the ERα-H antibody, two bands of apparent molecular weights of 67 and 54 kDa were detected, while when using the ERα-S antibody only the 67 kDa bands was identified. According to the suppliers, the ERα-H antibody does not recognize the native ER in the inactive 8S but reacts efficiently with the activated 4S receptor and thus it may be that both the 67 and 54 kDa variants are activated forms of the ERα protein.

Although studies have shown the expression of ERβ messenger RNA (mRNA) in various tissues, the expression of this ER isoform has not been studied extensively at the protein level in placentae using Western blotting methodologies. In our study, four discrete bands were detected for ERβ with apparent molecular weights of 76, 59, 54 and 41 kDa. The 59 kDa band has been reported as the full length ERβ [24-26] while the 54 kDa [12,14] and the 41 kDa bands, may be truncated variants of the ERβ isoform [18,24,27]. On the other hand, the higher molecular weight 76 kDa protein band, may indicate that posttranslational modification, such as phosphorylation [28,29] or glycosylation, [30,31] of the ERβ protein has taken place.

The smaller molecular weight bands, which were seen when using the ERα-H and ERβ antibodies, could be variants caused by alternative splicing, ER degradation products or proteins that are not related to ER but cross-react with the antibody. The low molecular weight bands were not detected in maternal whole brain or maternal uterus homogenates (data not shown) indicating that they are tissue specific. As these truncated forms were also not seen in non-pregnant uterus [32], then they may result from mechanisms unique to uterine stroma during pregnancy. In mice it has been shown that estradiol induces proteinase activities that degrade ER from a 65 kDa molecule to a 54 kDa variant [33]. Thus, the level of ER existing in target cells is dynamic and is probably under fine control by rapidly turning over proteinases, signifying an important posttranslational regulatory mechanism [32,33]. Posttranslational modification of ER could therefore be a common, albeit not appreciated, mechanism by which a target cell may regulate its responsiveness to estradiol.

There was a clear decline in ERα protein expression when the antibody that was raised against the hinge region was used. In fact the abundance of both the ERα variants (67 and 54 kDa) decreased significantly between 16 and 19 dg. Telleria *et al*. [22] has also shown that the antibody ER-715, raised against the hinge region of the rat, detected two bands 67 and 61 kDa; the 67 kDa variant was highly expressed at mid-pregnancy but barely detectable in early and late gestation while the 61 kDa variant remained unchanged. When using the antibody that was raised against the steroid binding domain, a significant decrease in the ERα protein expression was not detected, but there was a trend for the levels to decrease by 19 dg. The inability to reach significance may be due to the fact that this antibody is recognizing both the active and inactive receptors whereas the ERα-H recognized only the activated receptor, which is down-regulated as shown by the decrease in levels between 16 and 19 dg. Thus, the results using the ERα-S, which detects the total (inactive and active) receptor content, are influenced by the presence of the inactive receptor form which may not be down-regulated with gestation thereby biasing the overall result.

The decrease in abundance of the 59 and 54 kDa ERβ variants between 16 and 19 dg clearly indicate that there is down-regulation of the receptors. Although the 76 kDa variant showed a tendency to decrease at 19 dg this was not statistically significant. On the other hand, the 41 kDa ERβ variant showed a different pattern of expression with a tendency to increase between 16 and 19 dg but this also failed to reach statistical significance. The 41 kDa ERβ variant is probably derived from the native 59 kDa moiety as it wasn't extensively expressed and yet became the dominant form as the levels of the native ER decreased and it may be one of many forms of degradation fragments that happened to contain the anti-ERβ epitope. These thus far unreported bands may be tissue specific and may have certain yet unidentified functions in the placenta.

It has been shown by several investigators that total estrogen concentrations in blood rise markedly during human [34-36] pregnancies. In addition, we have shown that estradiol levels rise during pregnancy from 33 pg/ml at 16 dg to 39 pg/ml at 21 dg (unpublished data). The decrease in ER protein expression between 16 and 19 dg may be due to down-regulation of the receptors by the high concentration of estradiol in pregnant dams towards the end of gestation [37,38] since down-regulation of hormone receptors by their ligands has been reported for steroid hormones [37,39]. This decrease in ER expression near parturition suggests that placentae are relatively unresponsive to estrogens at this stage, which agrees with studies shown by others on bovine [40] and human [41] placenta. In order for down-regulation to take place, high levels of estradiol concentrations ≥ 0.5 mg/ml are required [37]. It has been reported by Sheehan *et al *[42] that a 5.0 mg/ml estradiol implant produces a steady serum estradiol level of 200 pg/ml and hence 0.5 mg/ml, which would produce an equivalent of 20 pg/ml serum estradiol, will be sufficient to cause down-regulation of ER.

Progesterone decreases the quantity of uterine ER by interfering with the replenishment of cytoplasmic ER thus decreasing the sensitivity of the tissue to estrogen [43]. Reports have confirmed that progesterone lowers the levels of nuclear ER when given in the presence or absence of estradiol [39]. In a previous study, we have shown that at 16, 19 and 21 dg the levels of maternal serum estradiol were 33, 29 and 39 pg/ml, respectively and levels in the amniotic fluid were 117, 157 and 226 pg/ml, respectively [44]. Furthermore, levels of progesterone in maternal serum at 16, 19 and 21 dg were 107, 84 and 40 ng/ml, respectively while in amniotic fluid the levels were 3, 3 and 4 pg/ml, respectively [44]. Thus the high levels of estradiol and progesterone in both maternal serum and amniotic fluid would therefore be a contributing factor to the down-regulation of the estrogen receptors as pregnancy advanced.

It is postulated that estradiol may have a specific growth-retarding effect on the placenta [45,46] that secondarily limits fetal growth [47,48] and therefore, minor elevations of maternal estradiol can be fatal to the embryo. Inhibitory action of estradiol may relate to its ability to induce transforming growth factor mRNA [49] which in turn encodes a protein that promotes apoptosis in decidualized endometrial stromal cells [50]. Indeed, the identification of ER in rat placentae suggests that they are target cells of estrogens. However, the decrease in estrogen receptor protein expression with pregnancy suggests that the placenta becomes unresponsive to estrogens pointing towards a protective role played by the placenta to 1) minimize the growth retarding effects of estradiol and 2) minimizing the possible deleterious effects of estrogens on the developing fetus. However, further studies on the function of ER proteins in placenta will provide a better understanding on their role during pregnancy.

## Conclusion

In this study, both ERα and ERβ isoforms and variants were detected from 16 dg. The increase in sex steroid hormone levels, reported with progression of pregnancy, leads to down-regulation of the ER. This down-regulation serves as a protective mechanism from the possible deleterious effects that high estrogen levels could have on the developing fetus.
